# Evaluation of Celastrol Antiviral Activity Against Equid Alphaherpesvirus Type 8 Infection

**DOI:** 10.3390/v17030347

**Published:** 2025-02-28

**Authors:** Yue Yu, Jiayu Wang, Lian Ruan, Li Chen, Muhammad Zahoor Khan, Anrong You, Changfa Wang, Liangliang Li, Huiying Ren, Tongtong Wang, Wenhua Liu

**Affiliations:** 1 College of Veterinary Medicine, Qingdao Agricultural University, Qingdao 266109, China; 2 Liaocheng Research Institute of Donkey High-Efficiency Breeding and Ecological Feeding, Liaocheng University, Liaocheng 252000, Chinazahoorkhan@lcu.edu.cn (M.Z.K.);

**Keywords:** EHV-8, celastrol, antiviral therapeutics, Nrf2/HO-1

## Abstract

Equid alphaherpesvirus type 8 (EHV-8) is a contagious pathogen that causes reproductive disorders, respiratory diseases, and viral encephalitis in equids, resulting in significant economic losses for the global horse and donkey industries. Currently, there are no approved antiviral drugs or vaccines available for EHV-8 control. In this study, we investigated the antiviral efficacy of celastrol against EHV-8 both in vitro and in vivo. Our results demonstrated that celastrol significantly inhibited EHV-8 infection in Rabbit kidney (RK-13) and equine dermal cells (NBL-6) in a dose-dependent manner. Mechanistic studies revealed that celastrol interfered with viral replication at multiple stages of the infection cycle. Furthermore, we found that celastrol induced an antiviral interferon response through activation of the Nrf2/HO-1 signaling pathway. Importantly, celastrol treatment significantly reduced EHV-8 replication and ameliorated lung pathology in a mouse model. These findings suggest that celastrol may represent a promising therapeutic agent for the treatment of EHV-8 infections.

## Simple Summary

Equid alphaherpesvirus type 8 (EHV-8) is an important pathogen that causes significant respiratory and neurological diseases in equids. Currently, limited therapeutic options exist to control EHV-8. In the present study, we demonstrated that celastrol significantly inhibited EHV-8 replication in RK-13 and NBL-6 cells in a concentration-dependent manner and at multiple stages of viral replication. Mechanistically, we found that celastrol induced IFN-α-related antiviral gene expression through activation of the Nrf2/HO-1 signaling pathway. It also reduced viral replication and ameliorated virus pathogenesis in a mouse lung model. These results suggest that celastrol could serve as a novel potential antiviral agent against EHV-8.

## 1. Introduction

Equid alphaherpesvirus type 8 (EHV-8) is a common pathogenic virus that primarily causes respiratory disease, reproductive disorders and neurological disease in equids [1]. It poses a significant and persistent threat to the global horse and donkey industries [2,3]. EHV-8 was first identified in 1988 in Australia, where it was isolated from the nasal cavity of an infected donkey [4]. In recent years, several outbreaks of EHV-8 have been reported in large-scale donkey farms across China, raising concerns regarding its increasing impact. For instance, Wang et al. isolated EHV-8 from an aborted fetus at a large-scale donkey farm in Liaocheng, China [5], and subsequently identified the virus in donkeys exhibiting neurological symptoms at another farm in Shandong Province [6]. Furthermore, an extensive epidemiological study conducted by Wang and colleagues reported an EHV-8 infection rate of approximately 38.7% (457/1180) across donkey farms in Shandong Province, underscoring the widespread nature of the virus and its significant threat to the donkey industry in China [7]. Despite these alarming observations, no effective antiviral drugs or vaccines are currently available for the control of EHV-8.

Celastrol, a natural compound derived from *Tripterygium wilfordii* Hook F [8,9], is known for its broad spectrum of biological activities [10,11,12,13] and has shown antiviral potential against various viruses. For example, Youn et al. demonstrated that celastrol inhibited HIV-1 Tat-induced pro-inflammatory cytokine expression through modulation of the MAPK and NF-κB signaling pathways [14]. Similarly, Fuzo et al. reported that celastrol suppressed SARS-CoV-2 replication in human and monkey cell lines, reducing interleukin-6 (IL-6) secretion [15]. Moreover, Yu et al. demonstrated that celastrol inhibits dengue virus replication by upregulating type I interferons and activating the downstream Jak-STAT signaling pathway [16]. Tseng et al. observed that celastrol suppressed hepatitis C virus replication in hepatoma cells via upregulation of heme oxygenase-1 (HO-1) through the JNK/MAPK/Nrf2 signaling cascade [17]. Additionally, Chen et al. reported that celastrol, acting as a potent HSP90 inhibitor, suppressed hepatitis C virus translation and inflammation in C57BL/6J mice by inhibiting heat shock protein 90β [18]. Despite these promising results, limited research has been conducted on the potential anti-EHV-8 activity of celastrol.

The aim of this study was to evaluate the antiviral efficacy of celastrol against EHV-8 and to elucidate the underlying mechanisms. Our results demonstrated that celastrol significantly inhibits EHV-8 replication in RK-13 and NBL-6 cells. Moreover, celastrol exerts its inhibitory effects at multiple stages of the EHV-8 replication cycle. Notably, celastrol significantly reduced EHV-8 replication in lung tissue of infected BALB/c mice. Taken together, our findings suggest that celastrol may serve as a promising therapeutic candidate for the control of EHV-8 infections.

## 2. Materials and Methods

### 2.1. Cells, EHV-8 Strains, and Reagents

Rabbit kidney cells (RK-13) and E. Derm cells (NBL-6) were cultured in Modified Eagle’s medium (MEM, Gibco, Fort Worth, TX, USA) supplemented with 10% fetal bovine serum (FBS, LONSERA, Ciudad de la Costa, Uruguay) and 1% penicillin–streptomycin (Beyotime, Nanjing, China) at 37 °C and 5% CO_2_. The EHV-8 SDLC66 strain (GenBank: MW816102.1), SD2020113 (GenBank: MW822570.1), and donkey/Shandong/10/2021 (GenBank: OL856098.1) were propagated in RK-13 cells. Celastrol was purchased from Sparkiade Biotechnology Co. Ltd. (Jinan, China) and dissolved in dimethyl sulfoxide (DMSO) (Solarbio, Beijing, China), and the final concentration is 1 mM.

### 2.2. Cell Viability Detection

Cytotoxicity of celastrol in EHV-8-susceptible cells was assessed using the cell counting kit-8 (cck-8) assay, as previously described [19]. Briefly, the RK-13 or NBL-6 cells were seeded into 96-well plates (1 × 10^4^ cells/well) and incubated with celastrol at various concentrations: 0 (containing 0.32%DMSO), 0.2 (containing 0.02%DMSO), 0.4 (containing 0.04%DMSO), 0.8 (containing 0.08%DMSO), 1.6 (containing 0.16%DMSO), and 3.2 (containing 0.32%DMSO) μM for 24 h. Then, 10 µL cck-8 reagent was added into each cell well and incubated at 37 °C for 2 h. Finally, the cells viability was assessed at 450 nm by Micro-Volume Spectrophotometer (Epoch, BioTek, Winooski, VT, USA) and analyzed using the formul: cell survival rate (%) = [OD (sample) − OD (blank)/OD (control) − OD (blank)] × 100%.

### 2.3. TCID_50_ Detection

The viral progeny titer was quantified by the 50% cell culture infectious dose (TCID_50_) method, as described previously [20]. Briefly, the RK-13 cells were seeded into 96-well plates with 1 × 10^4^ cells each well and cultured at 37 °C overnight. Viral supernatants were collected at designated time points, serially diluted by a factor of 10, and 100 μL of each dilution was added to eight replicate wells. The cells were incubated at 37 °C for 3–5 days, and the cytopathic effect (CPE) was observed daily. TCID_50_ values were calculated using the Reed–Muench method.

### 2.4. RNA/DNA Extraction and qPCR Analysis

Total RNA was extracted from cell samples using TRIzol reagent (Invitrogen, Carlsbad, CA, USA). One microgram of RNA was reverse-transcribed to cDNA using the PrimeScript II 1st Strand cDNA Synthesis Kit (Takara Bio, Beijing, China). Real-time PCR was performed on a StepOnePlus system to assess EHV-8 gD gene expression using specific primers (Table S1). GAPDH mRNA expression was used as an internal reference. Quantification of target gene expression was carried out using the 2^−ΔΔCt^ method [21].

For viral DNA quantification, the supernatant viral DNA was extracted using the TIANamp Virus DNA/RNA Kit (Tiangen, Beijing, China). An absolute quantification PCR (qPCR) assay was carried out to detect the supernatant DNA copies of EHV-8 with recombinant plasmids pMD18-T-gD and using the *ORF72*-F and *ORF72*-R primers as previously described [22] and calculated by normalization to the standard curve.

### 2.5. Western Blot Assay

Cellular samples were harvested at the indicated time points and lysed using RIPA lysis buffer (Solarbio, Beijing, China). The lysates were mixed with 5×SDS-PAGE loading buffer (Servicebio, Wuhan, China) and separated via 12% SDS-PAGE gels. Whereafter, the proteins were transferred onto polyvinylidene difluoride (PVDF) membranes (Merck Millipore, Billerica, MA, USA). The membranes were blocked with 5% nonfat dry milk, then incubated with primary antibodies: mouse anti-EHV-8 gD (made in our lab) and mouse anti-β-actin antibodies (Servicebio, Wuhan, China). After washing with PBS-T, the membranes were incubated with HRP-conjugated goat anti-mouse secondary antibody (Invitrogen, Shanghai, China). Protein bands were visualized and imaged using the ChemiDoc MP Imaging System (BioRad, Hercules, CA, USA).

### 2.6. The Viral Infection Inhibition Analysis

RK-13 and NBL-6 cells were seeded in 12-well plates, respectively, and pre-treated with celastrol at various dosages (0, 0.25, 0.5, and 1 μM) for 2 h and infected with EHV-8 SDLC66 at 0.1 multiplicity of infection (MOI) for 1 h. Following infection, the cells were cultured in 3% FBS MEM containing celastrol at indicated concentrations. Cellular supernatants were harvested at 24 h post infection (hpi) for progeny virus generation using TCID_50_ or qPCR assay. Meanwhile, these cells were collected to determine EHV-8 replication via Western blot assay according to the above description.

### 2.7. Indirect Immunofluorescence Assay (IFA)

IFA was performed to evaluate the antiviral efficacy against various EHV-8 strains as described previously [23]. The glass coverslips were pre-incubated into 12-well cell plates, then NBL-6 cells at 1 × 10^5^/well were seeded overnight, respectively. These cells were incubated with celastrol at various concentrations (0.25 μM, 0.5 μM, and 1 μM) for 2 h. Subsequently, these cells were infected with different EHV-8 strains, including SDLC66, SD2020113, or Donkey/Shandong/10/2021 at 0.1 MOI for 1 h and then changed with 3% FBS MEM containing celastrol at an indicated dosage. These cells were fixed with paraformaldehyde at 36 hpi and permeabilized with Triton X-100 at room temperature and washed with PBS. Subsequently, all cells were treated with mouse anti-EHV-8-positive serum (prepared in our lab) as primary antibody and incubated with the DyLight 594-conjugated goat anti-mouse IgG antibody as the secondary antibody. Finally, the coverslips were mounted onto slides with ProLong^®^Gold Antifade Reagent containing 4′,6-diamidino-2-phenylindole (DAPI) (Thermo, Waltham, MA, USA) and visualized using a DMi8 microsystems (Leica, Germany).

### 2.8. Time Course Analysis of Celastrol’s Activity Against EHV-8

To determine the specific phase of the EHV-8 life cycle affected by celastrol, NBL-6 cells were cultured in 12-well plates and treated with celastrol (1 μM) and EHV-8 SDLC66 (0.1 MOI) under different treatment conditions, including all stages, pre-treatment, co-treatment, and post-treatment. Subsequent to incubation, these cells were collected at 24 hpi to detect viral replication using quantitative PCR (qPCR) and Western blot detection.

### 2.9. Direct Virus Inactivation Assay

To investigate whether celastrol interacts directly with EHV-8, NBL-6 cells were seeded in 12-well plates and incubated overnight. We incubated EHV-8 SDLC66 (0.1, 0.5, and 1 MOI) with celastrol (1 μM) or DMSO for 2 h at 37 °C. The mixture was then added to the cells at 37 °C for 1 h. After incubation, the medium was replaced with 3% FBS MEM, and cells were cultured at 37°. Following incubation, the cells were harvested at 24 hpi for the detection of viral replication using qPCR and Western blot analysis.

### 2.10. siRNA Knockdown Assay

NBL-6 cells were pre-seeded into 12-well plates and transfected with siRNA targeting Nrf2 (siNrf2), siRNA targeting HO-1 (siHO-1), or an siRNA negative control (siNC) for 10 h before treatment with celastrol (1 µM) for 2 h (The sequence of siRNAs have shown in Table S2.), then infected with 0.1 MOI EHV-8 SDLC66. These cells were collected to detect EHV-8 replication using Western blot at 24 hpi.

### 2.11. Anti-EHV-8 Efficacy Assay in Vivo

Specific pathogen-free (SPF) male BALB/c mice (n = 12) were purchased from Pengyue (Jinan, Shandong Province, China). The mice were randomly divided into four groups (n = 3 per group): Group 1 (Mock group) received intraperitoneal injection administration of 100 µL MEM containing 2.5% DMSO; Group 2 (DMSO+EHV-8 infected group) received intraperitoneal injection administration of 100 µL MEM containing 2.5% DMSO; Group 3 (2 mg/kg Celastrol + EHV-8 infected group) and Group 4 (4 mg/kg Celastrol + EHV-8 infected group) were administered celastrol intraperitoneal injection in 100 µL MEM at day-1 (pre infection), and days 1, 3, and 5 post infection (dpi). Groups 2, 3, and 4 were subsequently received intranasal inoculation with 100 µL of EHV-8 at 1 × 10^5^ PFU (Plaque-Forming Units) per mouse. The mice were provided ad libitum access to food and water, and groups were housed in separate cages to prevent cross-contamination. Clinical symptoms were monitored daily. At 8 dpi, mice were euthanized by cervical dislocation, and lung tissues were collected and fixed in 10% neutral buffered formalin for histopathological examination.

### 2.12. Histopathological Evaluation

The hematoxylin and eosin (H&E) staining methodology was performed to detect the protective effect of celastrol against EHV-8 in lung tissues of mouse model as in previous studies [20,21,24]. In brief, the lung tissues were fixed in a 10% formalin solution. Subsequently, the tissues were embedded in paraffin wax and further cut into 4 µm. Then, these sections were mounted onto glass slides carefully and subjected to the H&E staining according to the instructions. The stained results were observed using an inverted microscope (Leika, Wetzlar, Germany).

### 2.13. Statistical Analysis

Data were processed and analyzed using GraphPad Prism 8.0 (San Diego, CA, USA). Differences between groups were assessed using the unpaired Student’s *t*-test. Statistical significance was denoted as follows: * *p* < 0.05, ** *p* < 0.01, *** *p* < 0.001.

## 3. Results

### 3.1. The Detection of Celastrol’s Cellular Viability in EHV-8 Susceptible Cell

**Figure 1:**
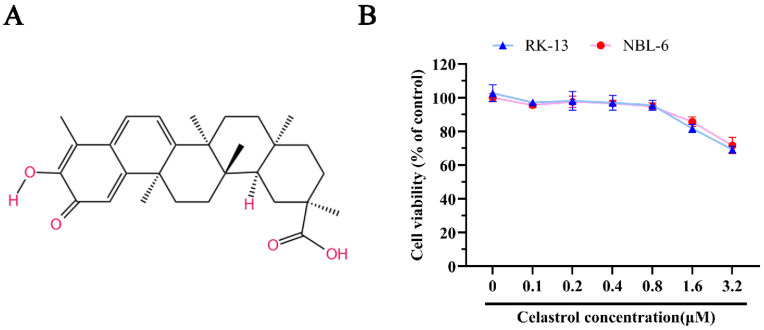
The chemical structure and cell cytotoxicity of celastrol. (**A**) The illustration of celastrol’s chemical structure. (**B**) The viability of celastrol in RK-13, and NBL-6 cells. These cells were seeded into 96-well cell plates, respectively, then incubated with celastrol at different concentrations (0.1, 0.2, 0.4, 0.8, 1.6 and 3.2 μM) for 24 h, the viability of the cells was detected by cck-8 kit. The relative cell viability of non-treated cells with celastrol served as blank control (set up as 100%).

The chemical structure of celastrol is illustrated in Figure 1A. To evaluate the potential cytotoxic effects of celastrol, cell viability assays were conducted using cck-8 in RK-13 and NBL-6 cell lines. These cells were treated with various concentrations of celastrol, followed by incubation with the cck-8 reagent. As shown in Figure 1B, celastrol concentrations up to 1 μM did not significantly affect cell viability in either RK-13 or NBL-6 cells, suggesting that celastrol does not exhibit substantial cytotoxicity at these doses.

### 3.2. Inhibition of EHV-8 Infection by Celastrol in Vitro

**Figure 2:**
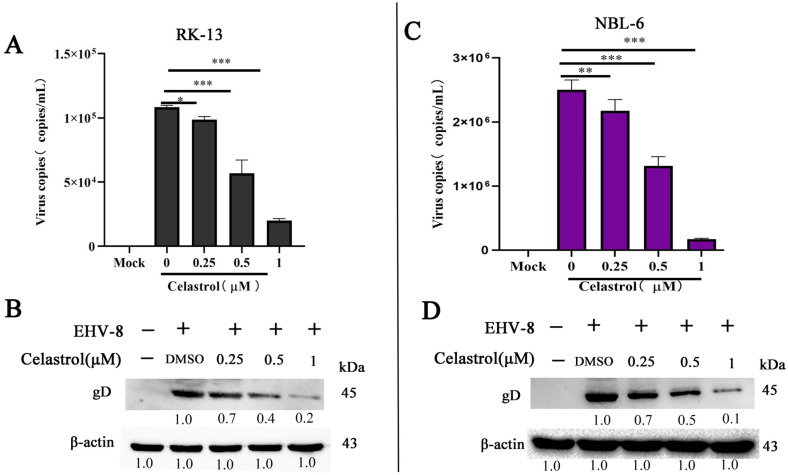
Celastrol exerts anti-EHV-8 activity in a dose-dependent manner. The RK-13 (**A**,**B**) and NBL-6 (**C**,**D**) cells were seeded into 12-well cell plates, then pre-treated with celastrol at different doses (0.25, 0.5, and 1 μM) for 2 h, and then incubated with EHV-8 SDLC66 at 0.1 MOI. These cell culture supernatants and cells were collected at 24 hpi to detect progeny virus generation and gD protein expression by qPCR and Western blot. The β-actin serves as a loading control. These data are presented as the mean ± SD of three independent experiments. * *p* < 0.05, ** *p* < 0.01, *** *p* < 0.001. The DMSO treated group served as 0 μM celastrol.

To assess the anti-EHV-8 activity of celastrol, RK-13 and NBL-6 cells were pre-incubated with celastrol at various concentrations for 2 h prior to infection with the EHV-8 strain SDLC66 (MOI 0.1) for 1 h. Cells were harvested at 24 hpi for subsequent analysis of viral replication by qPCR and Western blot. As shown in Figure 2A,B, celastrol decreased virus copies and gD expression in RK-13 cells. Similar reductions in gD expression were observed in NBL-6 cells (Figure 2C,D), indicating that celastrol inhibits EHV-8 replication in both cell lines.

### 3.3. Antiviral Activity of Celastrol Against Diverse EHV-8 Strains

**Figure 3:**
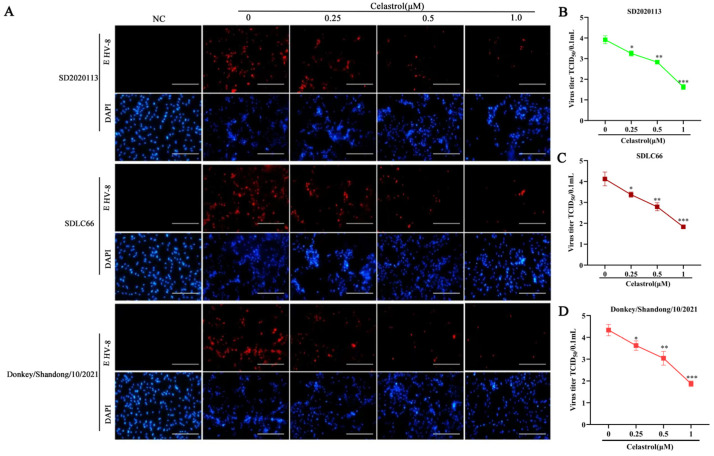
Celastrol possess a broad anti-viral activity against different EHV-8 strain. The NBL-6 cells were pretreated with celastrol at different dosages (0.25, 0.5, and 1 μM) or DMSO for 2 h, then infected with 0.1 MOI EHV-8 SD2020113, SDLC66, or Donkey/Shandong/10/2021, respectively, for 1 h. These cells were fixed at 36 hpi with paraformaldehyde to perform IFA (**A**). The mouse anti-EHV-8 positive serum (red) and DAPI (blue) were used to EHV-8 antigen and nucleocapsid stain. Images were captured using Leica DMi8 microsystems. Scale bar, 100 μm. The mock-infected cells were served as negative control. Meanwhile, the cellular supernatants were also harvested to detect the progeny viral titer by TCID_50_ (**B**–**D**). Data were presented as the means of normalized data ± standard deviations (error bars) based on at least three independent experiments. * *p*  <  0.05; ** *p*  <  0.01; *** *p*  <  0.001.

To determine whether celastrol’s antiviral effects extend to different EHV-8 strains, NBL-6 cells were pre-treated with celastrol at concentrations of 0.25 μM, 0.5 μM, and 1 μM for 2 h, followed by infection with three distinct EHV-8 strains: SD2020113, SDLC66, and EHV-8 donkey/2021 (MOI 0.1) for 1 h. After infection, the cells were cultured in 3% FBS MEM containing the indicated celastrol concentrations and fixed in paraformaldehyde at 36 hpi for IFA to detect EHV-8 antigens. As shown in Figure 3A, red fluorescent staining, indicative of viral antigen expression, was significantly reduced in a dose-dependent manner in cells treated with celastrol compared to untreated controls (0 μM). In parallel, supernatants were collected to assess progeny virus production by a TCID_50_ assay. In the TCID_50_ assay, typical cytopathic effects such as cell shrinkage and aggregation occurred in the cells, and the viral titers in celastrol-treated groups were significantly lower than those observed in the 0 μM celastrol-treated group, as shown in Figure 3B–D. These findings demonstrate that celastrol exerts broad-spectrum antiviral activity against multiple EHV-8 strains.

### 3.4. Celastrol Inhibits EHV-8 Infection at Multiple Stages

**Figure 4:**
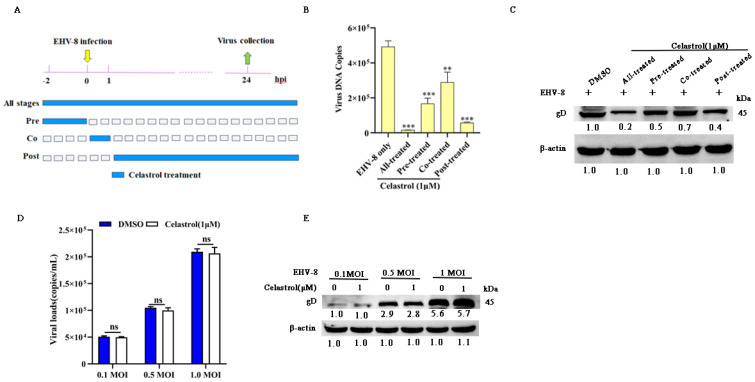
Celastrol reduces EHV-8 infection at multiple stages. Time-of-addition schematic (**A**). The NBL-6 cells were infected with 0.1 MOI EHV-8 SDLC66 and treated with celastrol at different time points, including All-stage treatment, Pre-treatment, Co-treatment, and Post-treatment. The NBL-6 cells were treated with celastrol (1 μM) as All-stage treatment, Pre-treatment, Co-treatment, and Post-treatment, whereafter, infected with EHV-8 SDLC66 at 0.1 MOI. These cell supernatants were collected to detect progeny virus generation by qPCR (**B**). Meanwhile, these cells were harvested to detect gD expression using Western blot (**C**). The NBL-6 cells were incubated with a mixture of EHV-8 SDLC66 (0.1, 0.5, and 1 MOI) and celastrol (1 µM) or DMSO, and the cellular supernatants and cells were collected 24 hpi to analyze the progeny viral copies (**D**) and gD expression (**E**) using qPCR and Western blot. β-actin acts as loading control. The data represent mean  ±  SD from three independent experiments. ** *p* < 0.01; *** *p*  <  0.001; ns: no significant.

To investigate the specific stage of the EHV-8 replication cycle affected by celastrol, we performed a time-course analysis in NBL-6 cells. Four experimental groups were designed as shown in Figure 4A: All-stage, representing the celastrol-treated group; Pre, representing the celastrol-pretreated group; Co, representing the celastrol and EHV-8 co-treated group; and Post, representing the celastrol post-treated group. The cellular supernatants and cells were collected at 24 hpi to assess EHV-8 replication by qPCR and Western blot. Our results demonstrated a significant reduction in gD protein expression in the Pre, Co, Post, and All-stage groups treated with celastrol compared to the DMSO-treated group (Figure 4B). A similar trend in gD protein expression was observed in Figure 4C. Notably, the All-stage celastrol treatment showed the minimal viral replication.

To assess whether celastrol could directly inactivate EHV-8 in NBL-6 cells, we incubated EHV-8 SDLC66 (0.1, 0.5, and 1 MOI) with celastrol (1 μM) or DMSO for 2 h at 37 °C. The mixtures were then inoculated into NBL-6 cells, and viral progeny production and gD expression were measured at 24 hpi by qPCR and Western blot. As shown in Figure 4D,E, no significant difference in viral progeny or gD expression was observed between the celastrol-treated and DMSO-treated infection groups, indicating that celastrol does not exhibit a direct virucidal effect on EHV-8 under the conditions tested.

### 3.5. Celastrol Induces Antiviral IFN Responses via Nrf2/HO-1 Activation

**Figure 5:**
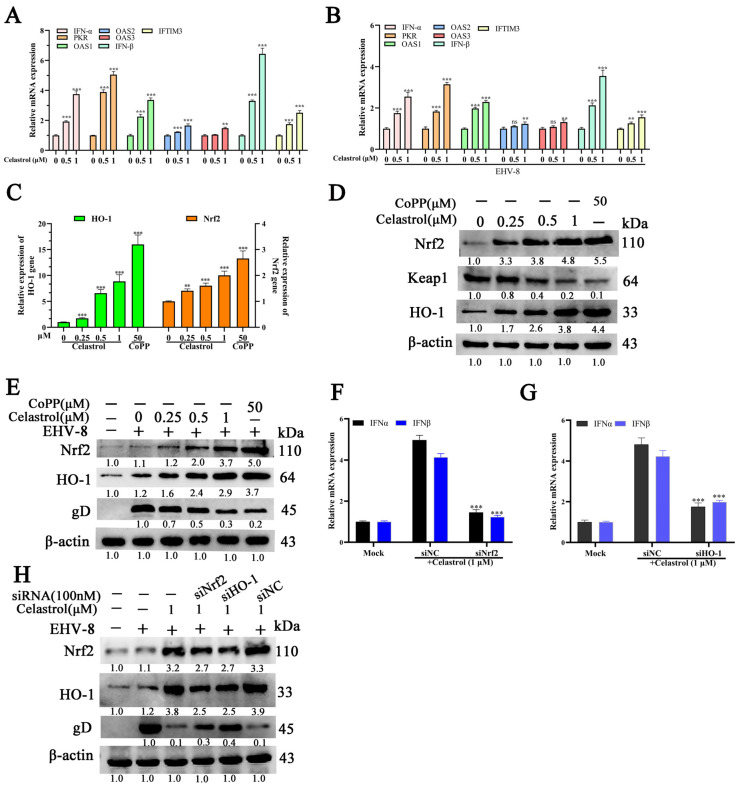
Celastrol exerts anti-EHV-8 activity Via Nrf2/HO-1 mediated IFN production. The NBL-6 cells were seeded into 12 well cell plates, and pretreated with celastrol at different concentrations (0, 0.5, and 1µM) for 2 h, and the mRNA expression of antiviral genes (IFN-α, IFN-β, PKR, OAS1, OAS2, OAS3, and IFITM3) were determined in NBL-6 cells without (**A**) or with (**B**) 0.1 MOI EHV-8 SDLC66 infection using qPCR. GAPDH served as the internal control. ** *p* < 0.01, *** *p* < 0.001, compared with 0 µM celastrol-treated cells. The NBL-6 cells were incubated with celastrol at different concentrations (0.25, 0.5, and 1 µM) for 24 h, and Nrf2 and HO-1 expression was determined by qPCR (**C**) and Western blot (**D**). The NBL-6 cells were incubated with celastrol at different concentrations (0.25, 0.5, and 1 µM) for 2 h and infected with 0.1 MOI EHV-8 SDLC66. These cells were collected to analyze the Nrf2 and HO-1 expression by Western blot (**E**). The CoPP served as the positive control. The NBL-6 cells were transfected with siNrf2, siHO-1, or siNC for 10 h and incubated with celastrol (1 µM) for 2 h, those cells were collected at 24 hpi to analyze the IFN-α/β expression in mRNA level using qPCR (**F**,**G**). GAPDH served as the internal control. ** *p* < 0.01; *** *p* < 0.001, compared with celastrol-treated cells. The NBL-6 cells were transfected with siNrf2, siHO-1, or siNC for 10 h and incubated with celastrol (1 µM) for 2 h and infected with 0.1 MOI EHV-8 SDLC66. Those cells were harvested at 24 hpi to detect Nrf2, HO-1, and viral gD protein expression using Western blot (**H**). β-actin served as the loading control. These data are presented as the mean ± SD of three independent experiments.

To investigate whether celastrol’s anti-EHV-8 activity is mediated through IFN-α upregulation, we analyzed the expression of IFN-α-related antiviral genes in NBL-6 cells using qRT-PCR and Western blot analysis. Celastrol treatment significantly enhanced the transcription of key antiviral genes, including *IFN-α*, *IFN-β*, 2′-5′-oligoadenylate synthetase 1 (*OAS1*), *OAS2*, *OAS3*, *PKR*, and *IFITM3*, in a dose-dependent manner in both EHV-8-infected and uninfected NBL-6 cells (Figure 5A,B).

Previous studies have demonstrated that the Nrf2/HO-1 signaling pathway regulates IFN-α-related antiviral responses [22,25,26,27]. To determine whether celastrol induces IFN-α-related antiviral gene expression through Nrf2/HO-1 activation, we quantified the protein levels of Nrf2, HO-1, and viral gD in celastrol-treated NBL-6 cells at varying concentrations. The results revealed significant upregulation of both Nrf2 and HO-1 (Figure 5C,D), accompanied by decreased viral gD protein expression (Figure 5E). To validate the role of Nrf2/HO-1 in celastrol-mediated antiviral responses, we transfected NBL-6 cells with Nrf2- or HO-1-specific siRNAs prior to celastrol treatment. Knockdown of either Nrf2 or HO-1 significantly attenuated IFN-α/β expression compared to the siRNA negative control (siNC) group (Figure 5F,G). Western blot analysis confirmed that Nrf2 or HO-1 silencing resulted in reduced Nrf2 and HO-1 protein levels and increased viral gD protein expression (Figure 5H). These results demonstrate that celastrol induces an antiviral IFN response against EHV-8 infection through activation of the Nrf2/HO-1 signaling pathway.

### 3.6. Celastrol Inhibits EHV-8 Infection in Vivo

**Figure 6:**
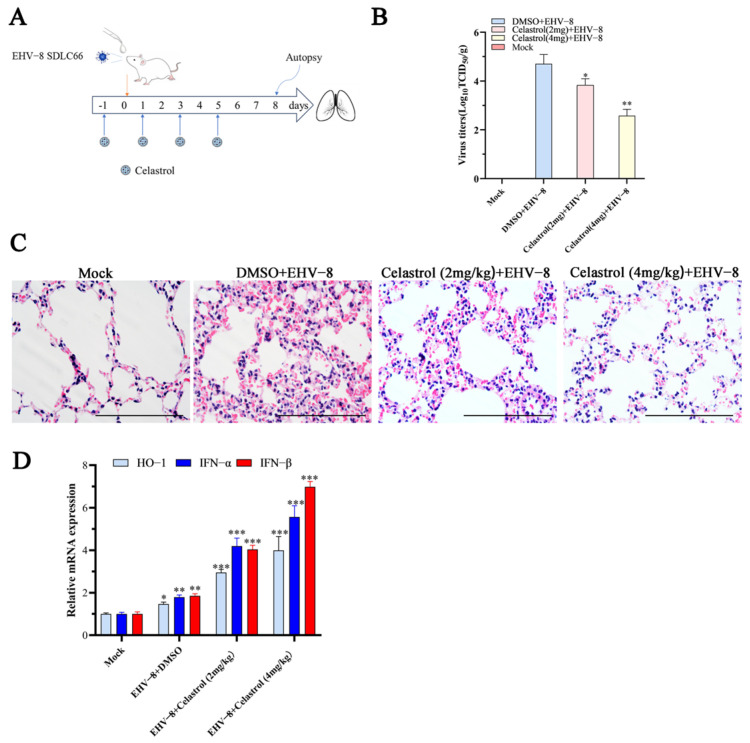
Celastrol decreases EHV-8 replication in mice. Twelve BALB/c mice were divided into four groups randomly: Mock group (only 2.5%DMSO treated); DMSO + EHV-8 infected group; Celastrol (2 mg/kg) + EHV-8 infected group; and Celastrol (4 mg/kg) + EHV-8 infected group. (**A**) The pattern diagram of the animal experiments. (**B**) The lung tissues of different groups were collected at 8 dpi to detect EHV-8 replication by TCID_50_. * *p* < 0.05; ** *p* < 0.01 compared with the DMSO + EHV-8 infected group. (**C**) Representative images of hematoxylin and eosin (H&E) staining in the lungs derived from different groups mice. Scale bar, 100 µm. (**D**) Total RNA was extracted from the lung tissues of different group mice at 8 dpi, and the mRNA expression levels of HO-1 and IFN-α/β were detected using qPCR. GAPDH serves as control. These data are presented as the mean ± SD of three independent experiments. * *p* < 0.05, ** *p* < 0.01, *** *p* < 0.001.

To evaluate the potential anti-EHV-8 properties of celastrol in vivo, we performed an animal experiment using BABL/c mice as shown in Figure 6A. To detect EHV-8 replication in the lungs of these mice at 8 dpi, the lung samples from different groups of mice were grinded and titrated on RK-13 cells, as depicted in Figure 6B. The mean viral titers in the DMSO+EHV-8 group at 5.01 × 10^4^ TCID_50_ were in contrast to the celastrol (2 mg/kg) + EHV-8 group, celastrol (4 mg/kg) + EHV-8 group where viral titers were obviously decreased, measuring at 6.3 × 10^3^ TCID_50_ and 3.8 × 10^2^ TCID_50_. Moreover, the histopathological detection of lung tissue specimens from the EHV-8 group unveiled severe alveolar wall thickening, resulting in the compression and collapse of alveolar cavities, accompanied by a marked infiltration of inflammatory cells. In contrast, lung tissues from celastrol-treated mice exhibited only minimal to mild alveolar wall thickening and reduced inflammatory cell infiltration (Figure 6C), suggesting that celastrol treatment alleviates lung tissue damage caused by EHV-8 infection. Additionally, qPCR analysis of lung tissue from the various treatment groups showed significantly higher expression levels of IFN-α, IFN-β, and HO-1 in the celastrol-treated groups compared to the DMSO-treated control group (Figure 6D). These findings suggest that celastrol exerts a protective effect against EHV-8 infection in vivo, potentially through the induction of IFN-α-related antiviral responses.

## 4. Discussion

**Figure 7:**
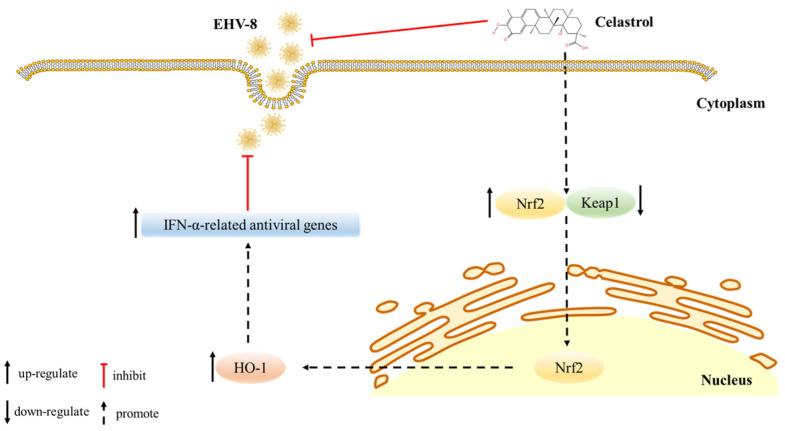
The Scheme diagram of the celastrol against EHV-8 infection mechanism. Celastrol inhibits EHV-8 infection at multiple stages in susceptible cells Via Nrf2/HO-1 signal axis activation, causing host cellular type I IFN response generation, and resulting in the inhibition of EHV-8 replication.

The rapid expansion of large-scale donkey farming in China has contributed substantially to local economic development. However, respiratory diseases and reproductive failures, particularly abortions, pose significant challenges to industry growth. The EHV-8 has been identified as a prevalent viral pathogen in donkey farms, causing respiratory illness, abortion, and viral encephalitis [1,6,7]. Despite its economic impact, therapeutic interventions for EHV-8 infections remain limited. In this study, we demonstrated that celastrol exhibits potent antiviral activity against EHV-8 both in vitro using susceptible cells and in vivo using a mouse model. Our mechanistic investigations revealed that celastrol mediates its antiviral effects through activation of the Nrf2/HO-1 signaling pathway, which subsequently enhances type I interferon production (Figure 7). These findings align with previous research showing that celastrol inhibits dengue virus (DENV) replication through the induction of IFN-α [16]. The conservation of this antiviral mechanism across different viral families suggests that celastrol may have broad-spectrum antiviral properties through modulation of host innate immune responses.

Celastrol, a pentacyclic triterpenoid isolated from *Tripterygium wilfordii* Hook F, has been reported to possess various pharmacological properties, including anticancer, anti-inflammatory, and antioxidant activities [28,29]. Previous investigations have established its antiviral efficacy against various viral pathogens, including HIV-1, HCV, SARS-CoV-2, and DENV [14,15,16,17]. In the present study, we demonstrated that celastrol exhibits potent antiviral activity against EHV-8 in both RK-13 and NBL-6 cell lines, with a clear dose-dependent relationship (Figure 2). The compound’s broad-spectrum efficacy was evidenced by its ability to suppress multiple EHV-8 strains with comparable potency (Figure 3). Mechanistic investigations revealed that celastrol interferes with multiple stages of the viral life cycle (Figure 4), suggesting a complex mode of action rather than targeting a single viral process.

The in vivo findings corroborated our in vitro observations, as celastrol administration significantly reduced viral burden in infected mice (Figure 6B) and markedly attenuated virus-induced pulmonary pathology (Figure 6C). Notably, the antiviral mechanism appears to involve both viral inhibition and immunomodulation, as evidenced by the enhanced expression of key antiviral factors including IFN-α, IFN-β, and HO-1 in both cell culture systems (Figure 5) and mouse lungs (Figure 6D). These findings suggest that celastrol’s therapeutic efficacy may be attributed to its dual ability to suppress viral replication while simultaneously potentiating host antiviral responses.

## 5. Conclusions

Altogether, this study demonstrates celastrol’s potent anti-EHV-8 activity through dual mechanisms: viral suppression and immune enhancement via IFN-α/β pathways. Celastrol reduces viral protein expression and viral titers in a dose-dependent manner, interfering with viral replication at multiple stages. Furthermore, the activation of the Nrf2/HO-1 signaling axis by celastrol leads to enhanced expression of antiviral genes, establishing a molecular basis for its therapeutic effects. Notably, in vivo studies revealed that celastrol administration not only attenuates viral load but also prevent tissue damage and enhances host immune responses. While these findings position celastrol as a promising therapeutic candidate against EHV-8, additional research is warranted to fully characterize its mechanism, pharmacokinetics, safety profile, and clinical efficacy in target populations.

## Data Availability

The original contributions presented in this study are included in the article. Further inquiries can be directed to the corresponding authors.

## Supplementary Materials

The following supporting information can be downloaded at: https://www.mdpi.com/article/10.3390/v17030347/s1, Table S1: The list of Primers used in this study; Table S2: The sequence of siRNA used in this study.
