# Supplementary material for: Evaluation of Celastrol Antiviral Activity Against Equid Alphaherpesvirus Type 8 Infection
Source: Viruses. 2025 Feb 28;17(3):347. doi: 10.3390/v17030347 (PMC11945448; doi:10.3390/v17030347)
Supplement: Supplementary file 1 [file viruses-17-00347-s001.zip › viruses-3449055-supplementary.pdf]

Table S1 The list of Primers used in this study

Genes	Forward primer (5′-3′)	Reverse primer (5′-3′)
ORF72	CCCACGTGTGCAACGCCTAT	ATACAGTCCCGAGGCAGAGT
IFN-α	TACTCAGCAGACCTTGAACCT	CAGTATTGGCAGCAAGTTGAC
IFN-β	AGCTCCAAGAAAGGACGA	GCCCTGTAGGTGAGGTTGATCT
OAS1	GGAGGCGGTTGGCTGAAGAGG	GAACCACCGTCGGCACATCC
OAS2	CCGGGCCAGTGCACAAGTTAG	CGATGGCACCGAGGACACC
OAS3	TCTGGGGTCGCTAAACATCAC	GATGACGAGTTCGACATCGGT
PKR	CGTTTCTTGCCTCCTGCTTTG	GGGACCTCCACATGACAGAAG
HO-1	AGTTCATGAAGAACTTTCA	TACCAGAAGGCCATGTCC
Nrf2	ATTCAATGATTCTGACTCTG	CGTATCCCCAGAAGAATGTA
GAPDH	GTCTCCTCTGACTTCAACAGCG	ACCACCCTGTTGCTGTAGCCAA

Table S2 The sequence of siRNA used in this study.

Primers	Sequences（5′-3′）
siHO-1-①	GGTCCTCACACTCAGCTTT
siHO-1-②	CCACCAAGTTCAAGCAGCT
siHO-1-③	CCACCAAGTTCAAGCAGCT
siNrf2-①	CTCCTTAAGAAGCAACTCA
siNrf2-②	GTCACTCTCTGAACTTCTA
siNrf2-③	GACATTCCCATTTGTAGAT
